# Utilization, Steering, and Spending in Vertical Relationships Between Physicians and Health Systems

**DOI:** 10.1001/jamahealthforum.2023.2875

**Published:** 2023-09-01

**Authors:** Anna D. Sinaiko, Vilsa E. Curto, Katherine Ianni, Mark Soto, Meredith B. Rosenthal

**Affiliations:** 1 Department of Health Policy and Management, Harvard T.H. Chan School of Public Health, Boston, Massachusetts; 2 Harvard PhD Program in Health Policy, Harvard University, Cambridge, Massachusetts

## Abstract

**Importance:**

Vertical relationships (eg, ownership or affiliations, including joint contracting) between physicians and health systems are increasing in the US.

**Objective:**

To analyze how vertical relationships between primary care physicians (PCPs) and large health systems are associated with changes in ambulatory and acute care utilization, referral patterns, readmissions, and total medical spending for commercially insured individuals.

**Design, Setting, and Participants:**

This case-control study with a repeated cross-section, stacked event design analyzed outcomes of patients whose attributed PCP entered a vertical relationship with a large health care system in 2015 or 2017 compared with patients whose attributed PCP was either never or always in a vertical relationship with a large health system from 2013 to 2017 in the state of Massachusetts. The sample consisted of commercially insured patients who met enrollment criteria and who were attributed to PCPs who were included in the Massachusetts Provider Database in 2013, 2015, and 2017 and for whom vertical relationships were measured. Enrollee and claims data were obtained from the 2013 to 2017 Massachusetts All-Payer Claims Database. Statistical analyses were conducted between January 5, 2021, and June 5, 2023.

**Exposure:**

Evaluation-and-management visit with attributed PCP in 2015 to 2017.

**Main Outcomes and Measures:**

Outcomes (which were measured per patient-year [ie, per patient per year from January to December] in this sample) were utilization (count of specialist physician visits, emergency department [ED] visits, and hospitalizations overall and within attributed PCP’s health system), spending (total medical expenditures and use of high-price hospitals), and readmissions (readmission rate and use of hospitals with a low readmission rate).

**Results:**

The sample of 4 030 224 observations included 2 147 303 females (53.3%) and 1 881 921 males (46.7%) with a mean (SD) age of 35.07 (19.95) years. Vertical relationships between PCPs and large health systems were associated with an increase of 0.69 (95% CI, 0.34-1.04; *P* < .001) in specialist visits per patient-year, a 22.64% increase vs the comparison group mean of 3.06 visits, and a $356.67 (95% CI, $77.16-$636.18; *P* = .01) increase in total medical expenditures per patient-year, a 6.26% increase vs the comparison group mean of $5700.07. Within the health care system of the attributed PCPs, the number of specialist visits changed by 0.80 (95% CI, 0.56-1.05) per patient year (*P* < .001), a 29.38% increase vs the comparison group mean of 2.73 specialist visits per patient-year. The number of ED visits changed by 0.02 (95% CI, 0.01-0.03) per patient year (*P* = .001), a 14.19% increase over the comparison group mean of 0.15 ED visits per patient-year. The number of hospitalizations changed by 0.01 (95% CI, 0.00-0.01) per patient-year (*P* < .001), a 22.36% increase over the comparison group mean of 0.03 hospitalizations per patient-year. There were no differences in readmission outcomes.

**Conclusions:**

Results of this case-control study suggest that vertical relationships between PCPs and large health systems were associated with steering of patients into health systems and increased spending on patient care, but no difference in readmissions was found.

## Key Points

**Question:**

Do vertical relationships between primary care physicians (PCPs) and large health systems change ambulatory and acute care utilization, referral patterns, readmissions, and spending?

**Findings:**

In this case-control study involving 4 030 224 observations, PCP–health system vertical relationships were associated with increased total specialist visits and total spending per patient-year as well as specialist visits, emergency visits, and hospitalizations within the health system. There was no change in readmission rates or use of hospitals with low readmission rates.

**Meaning:**

Findings of this study suggest that the vertical relationships between PCPs and large health systems were associated with steering patients into health systems and increased spending on patient care.

## Teaser

This case-control study compares health care use, cost, and delivery for patients before vs after their primary care physician enters a vertical relationship, including ownership, affiliation, and joint contracting, with a health system.

## Introduction

Vertical relationships between physicians and health systems have increased considerably over the past several years^1,2,3,4,5,6,7^ partly due to changes in health care payment and the increasing demand for coordinated care.^8,9,10,11^ Physician–health care system vertical relationships, which include ownership, affiliations, and joint contracting, may also form to increase referrals, to capture gains from shared administrative or capital resources, or to generate market power and negotiate higher payment rates from private insurers.^4,12,13^

There are both potential social benefits and adverse consequences to vertical consolidation.^14,15,16^ Physician–health system relationships may facilitate enhanced care coordination^17^ and communication among physicians and hospitals, which could play a role in lower duplicative testing or avoidable emergency department (ED) visits and thereby increase quality of care and decrease spending. Conversely, disrupting referral patterns could be associated with interrupted patient-physician relationships and lower quality.^18^ Moreover, as physician-hospital integration is known to be a factor in higher prices,^19,20,21,22,23,24,25^ it could also play a role in increased use of higher-cost physicians and facilities without gains in quality.

Vertical relationships in health care have garnered increased scrutiny from antitrust authorities and state regulators.^26,27^ However, evidence of the association of physician–health system relationships with care delivery patterns remains incomplete.^28^ Studies examining hospital-physician joint ownership, 1 type of vertical relationship, found a higher probability of patient use of facilities within the joint health system^18,21,29^ and shifting of care from nonhospital to hospital sites.^30,31^ In this study, we used a novel measure of physician–health system vertical relationship that represents progress over prior measures because it includes both ownership and joint contracting affiliations and does not rely on hospital self-reported data. The study objective was to analyze how vertical relationships between primary care physicians (PCPs) and large health systems, which include specialists and hospitals, are associated with changes in ambulatory and acute care utilization, referral patterns, readmissions, and total medical spending for commercially insured individuals in Massachusetts.

## Methods

The Harvard University Longwood Area Institutional Review Board deemed this case-control study exempt from review and granted a waiver of Health Insurance Portability and Accountability Act authorization. We followed the Strengthening the Reporting of Observational Studies in Epidemiology (STROBE) reporting guideline.

### Data Sources

We used 4 data sources. The eAppendix in Supplement 1 provides additional detail on these 4 data sources. First, we used the Massachusetts Health Quality Partners Massachusetts Provider Database (MPD) for 2013, 2015, and 2017. The MPD is a proprietary database linking physicians to practice sites, medical groups, and corporate or contracting affiliations with health systems in the state.^32^ The MPD collects data directly from provider organizations (defined as medical groups and health systems) rather than from payer records, and these data are updated and validated annually by approximately 95% of provider organizations using an online data validation tool. Second, we used the Center for Health Information and Analysis (CHIA) Massachusetts Hospital Cost Reports for 2013, 2015, and 2017 and the Relative Price data for hospitals for 2018, which reports mean hospital prices that accounted for differences in patient acuity, service mix, and payer mix.^33,34^

Third, we used 2017 hospital readmission rates from the Centers for Medicare & Medicaid Services (CMS) Hospital Quality Initiative Hospital Care Compare.^35^ Fourth, we used the 2013 to 2017 Massachusetts All-Payer Claims Database (APCD).^36^ The 2013 to 2015 APCD included enrollees and claims from all commercial health plans in Massachusetts; in 2016 to 2017, the APCD included all fully insured commercial plans and some self-insured plans, such as the largest commercial purchaser in the state.

### Study Population

The unit of analysis was the patient-year, defined as per patient per year from January to December in this sample. We selected patient-years wherein the patient was enrolled in 1 of 8 of the largest commercial insurance plans in the state (we excluded other insurance carriers due to lack of reliability of their claims),^37^ had at least 7 months of enrollment to observe most of the care during the year, and had at least 1 evaluation-and-management visit during the year with a physician in the MPD (eFigure 1 in Supplement 1).

We attributed each patient-year to the PCP in the MPD with the plurality of the patient’s evaluation-and-management claims for that year. Attribution to the PCP was done separately within each year to allow for patient changes in PCPs over time.^38,39^ When there was a tie in the plurality of claims between PCPs, we randomly attributed the patient-year to 1 of the PCPs in the tie. Patient-years with no evaluation-and-management claims with a PCP were attributed to another physician who was associated with the plurality of their evaluation-and-management claims (12.9% of attributed patient-years). Patient-years wherein the plurality of claims was with a physician who was not in the MPD (11.0% of patient-years) were reattributed to a PCP in the MPD with whom the patient had the most evaluation-and-management claims. We conducted a sensitivity analysis in which we excluded these patient-years and found similar results.

### Measure of Vertical Relationship 

In 2 steps, we developed a novel measure of a vertical relationship between a PCP and a large health system, which included other physicians and hospitals. First, we identified hospital ownership by any health system in 2013, 2015, and 2017 using the CHIA Massachusetts Hospital Cost Reports. Using methods that were described previously (eMethods in Supplement 1),^40^ we classified a health system as large if the share of total discharges from its hospitals was at least 20% of the health system’s geographic market (eTable 1 in Supplement 1).

Second, we ascertained whether each PCP had a vertical relationship with any of the large health systems in 2013, 2015, and 2017 by applying the physician–health system relationships in the MPD. This process captured full integration (ownership) and joint contracting or affiliation (eMethods in Supplement 1). For the 2 years in the study period for which we did not have MPD data, we applied the physician’s measure from the prior year (ie, 2013 measure in 2014, and 2015 measure in 2016). In sensitivity analyses, we used the Massachusetts Registration of Provider Organization program database to identify ownership relationships in 2015 or 2017, and the results were similar and slightly larger in magnitude (eMethods and eTable 5 in Supplement 1).

### Outcomes

For each patient-year, we measured utilization. Specialist physician visits were the count of patient-days with any claim (ie, evaluation and management or other) from a specialist physician, and ED visits were the count of patient-days with a claim indicating either the ED as the site of service or an evaluation-and-management code in the ED setting. Both inpatient and outpatient services were included in ED visits. We also counted hospitalizations using the admission dates on facility claims with inpatient hospital facility as the site of service, requiring a 30-day clean period between subsequent hospitalizations. Hospitalizations were attributed to the facility associated with the plurality of claims during the hospital episode.

We constructed a within-system utilization measure to identify steering, defined as how the amount of care received within a large health system changed after a physician entered into a vertical relationship with the health system. For each patient-year, we identified the largest health system with which the patient’s attributed PCP had a vertical relationship during the study period, if any. We ascertained the specialist visits, ED visits, and hospitalizations that occurred within this health system.

For example, if a patient was attributed to a PCP in 2013 to 2015, we identified the largest health system with which this PCP had a vertical relationship at any point between 2013 and 2017. Suppose that this PCP had a new affiliation with UMass in 2015. We calculated the patient’s use of specialist care, ED care, and hospitalizations with UMass hospitals and health care facilities and practitioners during 2013 to 2015. This period included years before and after the PCP was affiliated with UMass. The number of services received within system for patients whose attributed PCP was never in a vertical relationship with a large system was 0 in all years by construction. These patients were excluded from analyses of changes in within-system utilization.

To identify readmission rates, first we followed the methods outlined by CMS value-based payment programs^41^ to construct a patient-level indicator of any 30-day readmission within a given year. The 30-day readmission was defined as an unplanned readmission to an acute care hospital, for any cause, within 30 days of discharge from a short-stay acute care hospitalization. Second, we constructed terciles for low, medium, and high readmission rate hospitals in Massachusetts based on their 2017 hospitalwide, all-cause, 30-day readmission rates reported by CMS Hospital Care Compare.^35^

Additionally, we calculated spending. Total medical expenditures per patient-year were defined as the sum of the paid amount (eg, allowed amount) on the patient’s claims within the year; the sum was inflated to January 2022 dollars using the Consumer Price Index for urban consumers. Moreover, using the CHIA Relative Price data, we classified hospitals into terciles based on their statewide, cross-payer relative prices paid by commercial payers (eMethods in Supplement 1).^32^ For each hospitalization in this sample, we ascertained whether the attributed facility was in the highest-price tercile.

Other covariates included patient sex, age, and number of months enrolled in the plan within the year (ie, 7-12 months), which were extracted from the Massachusetts APCD. We identified patient clinical risk by counting the Hierarchical Condition Categories (HCCs) per patient-year; we used the HCCs from the individual and small group marketplace and from a commercially insured population younger than 65 years.^42,43^ We ascertained market concentration among physicians using the Herfindahl-Hirschman Index at the 3-digit zip code level (eMethods in Supplement 1).

### Statistical Analysis 

In this repeated cross-sectional analysis, we used variation in a PCP’s vertical relationship with a large health system from 2013 to 2017 to estimate the association between vertical relationships and study outcomes. To isolate this association, we excluded patients for whom the attributed PCP changed across years to a PCP in a different health system (12.9% of patient-years) because outcomes for these patients were associated with patient choice (ie, switching PCPs) and PCP vertical relationships. We excluded patient-years wherein PCPs changed health systems more than once during the study period (1.3% of patient-years) because we could not separately estimate the association between 2 vertical relationships and outcomes.

In this sample, PCPs formed vertical relationships in either 2015 or 2017. Given the time-varying treatment, we used a stacked event study design that accounted for heterogeneity over time and avoided bias from 2-way fixed-effect models.^44,45^ This study design compared changes in outcomes for patients in the treatment group before vs after their PCPs entered a vertical relationship with changes in outcomes for patients in the comparison group over the same period. In a stacked event study, comparing the pretreatment period of a later-treated group to the posttreatment period of an early-treated group is problematic, and thus we did not include in the comparison group those patients with PCPs who entered vertical relationships at other times during 2013 to 2017 as it could bias the estimates.^46,47,48^ The comparison group included only patients whose PCP was either never (never treated) or always (always treated) in a vertical relationship with a large health system from 2013 to 2017. Although there were concerns with using always-treated units in the comparison group,^49^ we included these observations (1) because the always-treated PCPs were plausibly more similar to the treatment group in that they also selected to join vertical relationships with health systems and (2) to allow inclusion of within-system utilization as an outcome in the analysis; because the never-treated PCPs were not in a vertical relationship with any health system, we could not measure within-system visits for patients of these PCPs. To assess the appropriateness of including the always-treated units, we tested and found support for parallel patterns in outcomes between the treatment and both comparison groups in the pretreatment period (eMethods and eFigure 2 in Supplement 1). In sensitivity analyses, we estimated the models using only the never-treated physicians as the comparison group, and the results were similar.

Models included PCP fixed effects, which controlled for unobservable, time-invariant characteristics of physicians (including those associated with patient choice), calendar year fixed effects, and experiment fixed effects (indicators for whether the observation was treated in 2015 or 2017). All models controlled for patient age, sex, their interaction, clinical health risk (0, 1, 2, 3, 4, or ≥5 HCCs), and market concentration. Because physicians are organized into medical groups, and entire medical groups may become vertically integrated at the same time, SEs were clustered at the medical group level.

From these models, we calculated the coefficient of interest, which was the mean change in the outcome associated with a patient’s attributed PCP becoming vertically integrated with a large health system. We used unpaired, 2-tailed *t* tests and a threshold of 2-sided *P* = .05 to determine statistical significance. The eMethods in Supplement 1 provides additional detail. Statistical analyses were performed between January 5, 2021, and June 5, 2023, using Stata, version 16 (StataCorp LLC).

## Results

The study sample included 4 030 224 observations. Of these patients, 2 147 303 were females (53.3%) and 1 882 921 were males (46.7%), with a mean (SD) age of 35.07 (19.95) years, and 31.4% had at least 1 chronic condition (Table 1; eTables 2-4 in Supplement 1). Across all patient-years, 35.2% of patients’ attributed PCPs had a vertical relationship with a large health system, the mean (SD) annual medical spending was $5670.74 ($18 992.79), and the mean (SD) readmission rate was 5.66% (23.10%). Table 1 reports summary statistics on specialist visits, ED visits, and hospitalizations per patient-year, overall and within the attributed PCP’s treatment and comparison groups.

**Table 1.** Summary Statistics

Measure	Overall		Treatment group		Comparison groups			
					Always-treated PCPs		Never-treated PCPs	
	No. (%)	Mean (SD)	No. (%)	Mean (SD)	No. (%)	Mean (SD)	No. (%)	Mean (SD)
Observations	4 030 224 (100)	NA	152 194 (3.8)	NA	1 382 994 (34.3)	NA	2 495 036 (61.9)	NA
Patient’s attributed PCP has vertical relationship with a large health system^a^^,^^b^	1 419 847 (35.2)	35.23 (47.77)	37 988 (26.0)	24.96 (43.28)	1 382 994 (100)	100 (0)	0	0
Patient characteristics								
Age, y	NA	35.07 (19.95)	NA	37.56 (19.05)	NA	36.64 (19.56)	NA	34.05 (20.14)
Female sex	2 147 303 (53.3)	53.28 (49.89)	82 504 (54.2)	54.21 (49.82)	757 604 (54.8)	54.78 (49.77)	1 307 149 (52.4)	52.39 (49.94)
Male sex	1 882 921 (46.7)	46.72 (49.90)	69 690 (45.8)	45.79 (49.80)	625 390 (45.2)	45.22 (49.80)	1 187 887 (47.6)	47.61 (49.94)
With a chronic condition^c^	1 263 475 (31.4)	31.35 (46.39)	48 672 (32.0)	31.98 (46.64)	444 909 (32.2)	32.17 (46.71)	769 968 (30.9)	30.86 (46.19)
Total medical expenditures per patient-year, $	NA	5670.74 (18 992.79)	NA	5250.26 (16 722.93)	NA	6558.62 (22 781.72)	NA	5204.23 (16 651.46)
Any 30-d readmission rate, %	NA	5.66 (23.10)	NA	5.39 (22.58)	NA	6.20 (24.12)	NA	5.33 (22.47)
Total ED visits per patient-year	NA	0.36 (1.08)	NA	0.39 (1.13)	NA	0.35 (1.02)	NA	0.37 (1.11)
ED visits within system per patient-year^d^	NA	0.19 (0.55)	NA	0.11 (0.43)	NA	0.15 (0.52)	NA	0.36 (0.67)
Total specialist visits per patient-year	NA	3.04 (5.73)	NA	2.59 (5.32)	NA	3.52 (6.48)	NA	2.79 (5.28)
Specialist visits within system per patient-year^d^	NA	1.86 (4.46)	NA	1.25 (3.30)	NA	2.73 (5.63)	NA	1.10 (2.89)
Total hospitalizations per patient-year	NA	0.04 (0.25)	NA	0.04 (0.25)	NA	0.05 (0.27)	NA	0.04 (0.24)
Hospitalizations within system per patient-year^d^	NA	0.03 (0.21)	NA	0.01 (0.13)	NA	0.03 (0.22)	NA	0

Abbreviations: ED, emergency department; NA, not applicable; PCP, primary care physician.

^a^ This vertical relationship is defined as vertical integration or joint contracting between a physician medical group and a health system.

^b^ A health system was classified as large if the share of total discharges from its hospitals was at least 20% of the system’s geographic market, defined using the hospital referral region in 2017.

^c^ Chronic conditions were ascertained using the Hierarchical Condition Categories, developed for the Centers for Medicare & Medicaid Services Medicare Advantage program and used in the individual and small group marketplace to estimate medical expenditure risk based on patient diagnoses and demographic characteristics.

^d^ Within system refers to patient’s use of services in the health system with which their attributed PCP has a vertical relationship.

### Vertical Relationships and Care Utilization 

**Figure 1.**
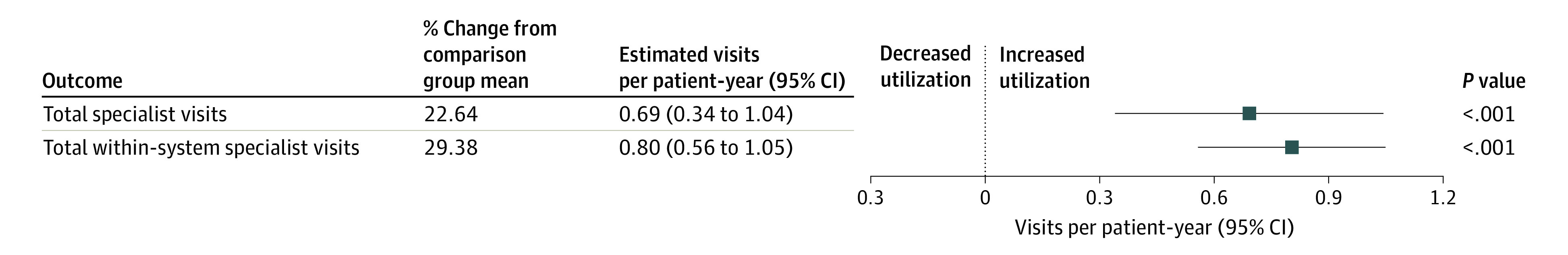
Association Between Vertical Relationships and Specialist Visits per Patient-Year Estimates with CIs that did not cross the vertical line at 0 were significantly different from 0. Error bars represent 95% CIs. The total specialist visit model consisted of 7 607 365 patient-years, and the total within-system model included 2 913 972 patient-years.

**Figure 2.**
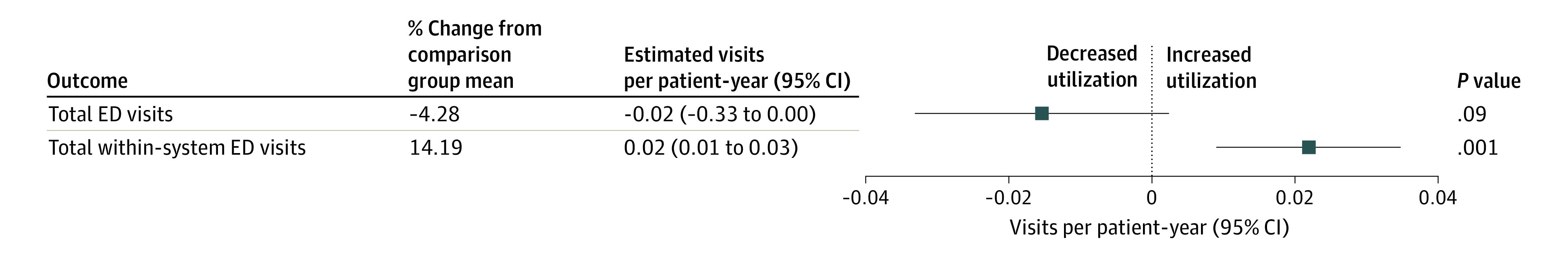
Association Between Vertical Relationships and Emergency Department (ED) Visits per Patient-Year Estimates with CIs that did not cross the vertical line at 0 were significantly different from 0. Error bars represent 95% CIs. The total ED visit model consisted of 7 607 365 patient-years, and the total within-system model included 2 913 972 patient-years.

**Figure 3.**
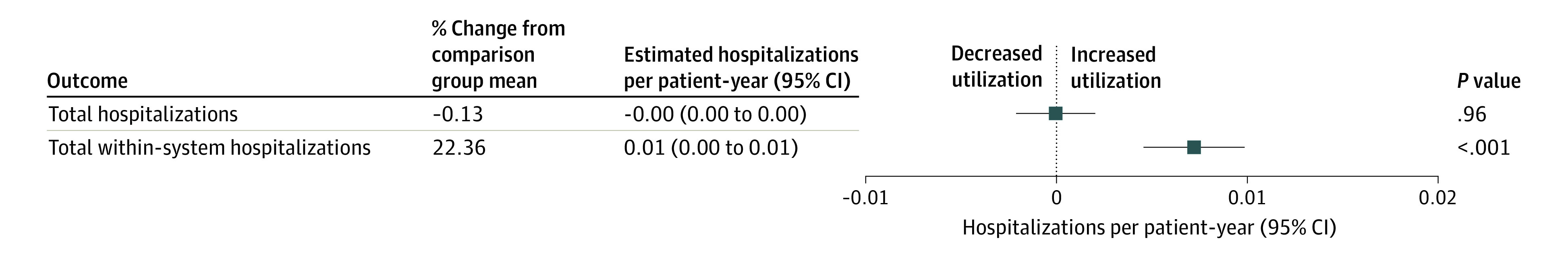
Association Between Vertical Relationships and Hospitalizations per Patient-Year Estimates with CIs that did not cross the vertical line at 0 were significantly different from 0. Error bars represent 95% CIs. The total hospitalization model consisted of 7 607 365 patient-years, and the total within-system model included 2 913 972 patient-years.

A patient’s PCP entering a vertical relationship with a large health system was associated with an increase of 0.69 (95% CI, 0.34-1.04; *P* < .001) specialist visits per patient-year, a 22.64% increase vs the comparison group mean of 3.06 specialist visits per patient-year (Figure 1). There were no statistically significant changes in total ED visits or hospitalizations (Figure 2 and Figure 3).

The PCP–health system vertical relationships were associated with an increase of 0.80 (95% CI, 0.56-1.05; *P* < .001) specialist visits per patient-year rendered within the PCP’s health system, a 29.38% increase from the comparison group mean of 2.73 within-system specialist visits per patient-year (Figure 1). Vertical relationships were associated with an increase in within-system ED visits per patient-year of 0.02 (95% CI, 0.01-0.03; *P* = .001), a 14.19% increase vs the comparison group mean of 0.15 ED visits per patient-year (Figure 2). Additionally, there was an increase of 0.01 (95% CI, 0.00-0.01; *P* < .001) within-system hospitalizations per patient-year, a 22.36% increase vs the comparison group mean of 0.03 hospitalizations per patient-year (Figure 3).

### Vertical Relationships and Spending and Readmissions

Total medical expenditures per patient-year increased $356.67 (95% CI, $77.16-$636.18; *P* = .01), a 6.26% increase vs the comparison group mean of $5700.07, after a PCP entered into a vertical relationship with a large health system (Table 2). The probability of admission to a high-price hospital in the comparison group was 55.75%; PCPs’ vertical relationships with a large health system were associated with a 4.91%-higher probability of admission to a high-price hospital when hospitalized (estimated coefficient, 0.03; 95% CI, −0.00 to 0.06; *P* = .06), but this finding was not statistically significant. There were no changes in the probability of readmission or the probability of admission to a hospital in the lowest all-cause 30-day readmission rate tercile associated with PCP–health system vertical relationships (Table 2).

**Table 2.** Association Between Vertical Relationships and Spending and Readmission Outcomes^a^

	Sample size, No.	Comparison group mean (2013-2017)	Estimated coefficient (95% CI)	*P* value	Percentage change from comparison group mean
**Spending outcomes per patient-year**					
Total medical expenditure, $	7 607 365	5700.07	356.67 (77.16 to 636.18)	.01	6.26
Probability of admission to a high-price hospital	266 461	0.55	0.03 (0.00 to 0.06)	.06	4.91
**Readmission outcomes per patient-year**					
Readmission rate	281 878	0.06	0 (−0.01 to 0.01)	.77	2.33
Probability of admission to a hospital with a low readmission rate	238 312	0.23	0 (−0.03 to 0.04)	.78	2.06

^a^ Estimated coefficients were based on stacked event study models, including patient characteristics; experiment, calendar year, and physician fixed effects; and the physician market Herfindahl-Hirschman Index of total patient spending at the level of the first 3 digits of a zip code.

## Discussion

Considering the continued growth in vertical relationships between physicians and health systems, it is critical to understand the risks and benefits of this type of consolidation. Using a novel measure that captures both joint ownership and affiliations, we explored the association of vertical relationships with changes in ambulatory and acute care utilization in Massachusetts. The dominant changes were those that we characterized as steering: specialist visits, ED visits, and hospitalizations rendered by the associated large health systems increased when the PCP became owned or affiliated with the health system. We also observed some potential inducement: the overall specialist physician visits increased, which raises the possibility of PCPs that are integrated with large health systems making a specialist referral, all else being equal. We found that PCP–health system vertical relationships were associated with increased total medical spending per patient-year, but there was no evidence to suggest that these vertical relationships had a role in reduced readmissions or in directing patients to hospitals with lower readmission rates.

Steering is not necessarily a factor in lower-quality care. It is possible that there is better coordination (eg, via shared medical records) and less redundancy when care across the continuum is delivered within a health system, which could be associated with higher quality and reduced costs. It is also possible that the overall increase in specialist visits was due to wider access to a large health system with specialists. However, the finding of no change in patient readmissions might suggest limited gains from increased coordination. Moreover, a national study of independent physicians becoming partially integrated with a hospital found no change in process measures of quality but an increase in postprocedure complications.^11^ Other national studies have found little to no changes in hospital-level outcomes following hospital-physician ownership.^24,50^ Some studies have found small, beneficial associations between vertical integration and quality, although these studies were limited by regional samples and observational design.^51,52^ Overall, vertical relationships appeared to be no panacea to health care access or coordination.

It is also possible that some visits are directed away from lower-quality or higher-cost physicians or hospitals. While we did not measure the comparative quality of downstream care aside from readmission rates, its potential cost-lowering benefits are unsupported by our findings on medical spending and other evidence that the price of physician visits in vertically integrated systems is higher than that in independent practices.^23,40^

While we reported mean associations across Massachusetts, these associations likely vary by system and market characteristics. Health care organizations that attempt to clinically integrate physicians into their systems using organizational strategies may have differential associations with care utilization (including steering) and quality measures.^53,54^ It is also likely that the observed associations vary with the strength of the financial incentives between physicians and health systems, as stronger incentives may lead to increased clinical integration. Health care organizations that accept risk contracts (eg, accountable care organizations and bundled payment arrangements) may be more likely to steer patients inside the health system when the care provided there is expected to be cost-saving or quality-improving.^55,56^ We were unable to test for this variation because the data set we used did not include measures of financial incentives or risk contracts.

### Limitations

This study has several limitations. It focused on 1 state over a 5-year period, which may limit the generalizability of findings to other parts of the US and to longer study duration. However, Massachusetts has several large health systems competing alongside smaller health systems and independent physicians, and many large metropolitan areas in the US have a similar configuration of health care professionals. Although the duration of time required to see changes in care utilization and steering was unclear, we expected that 5 years was sufficiently long to observe, at a minimum, the start of these changes. We used within-physician changes in vertical relationships to estimate associations, but these estimates could be influenced by other marketwide changes affecting the same physicians undergoing vertical consolidation. Due to data limitations, there was likely measurement error in determining whether a PCP was in a vertical relationship in 2014 and 2016, which may bias these results toward the null. These results reflect the associations of 2 types of vertical relationships (ownership relationships and affiliations that include joint contracting), and we did not distinguish between ownership and affiliation.

## Conclusions

This case-control study showed that vertical relationships between PCPs and large health systems were associated with steering patients to health systems and increased spending on patient care. These findings raised concern that the steering of care corresponded with insurers paying more for the same types of care visits and that this form of consolidation may be associated with overall higher costs. Moreover, we found that vertical relationships were associated with increased specialist visits within large health systems, which warrants further study to ascertain whether these visits represent low-value care or improved access to specialists. Policymakers, regulators, and purchasers may need to consider adopting a portfolio of countermeasures to limit the adverse implications of vertical relationships for the total cost of care. These countermeasures include antitrust enforcement, adoption of transparency and patient steering tools that encourage patients to seek care from lower-cost physicians and hospitals, and alternative payment models that reward use of lower-priced care.

## Notes

Supplement 1. **eAppendix.** Data Information
eMethods.
**eTable 1.** Classification of Health Systems **eTable 2.** Hospitalization Characteristics **eTable 3.** Patient-Year Characteristics **eTable 4.** Physician Characteristics, Stratified by Treatment and Comparison Group **eTable 5.** Results from Sensitivity Analysis Using the RPO Data **eFigure 1.** Flow Chart of Patient-Year Observations Included in Analyses **eFigure 2.** Plots of Test of Differential Pre-Trends in Study Outcomes (Event Study Regression Results)

Supplement 2.
Data Sharing Statement
