# Supplementary material for: Utilization, Steering, and Spending in Vertical Relationships Between Physicians and Health Systems
Source: JAMA Health Forum. 2023 Sep 1;4(9):e232875. doi: 10.1001/jamahealthforum.2023.2875 (PMC10474555; doi:10.1001/jamahealthforum.2023.2875)
Supplement: Supplement 1. — eAppendix. Data Information eMethods. eTable 1. Classification of Health Systems eTable 2. Hospitalization Characteristics eTable 3. Patient-Year Characteristics eTable 4. Physician Characteristics, Stratified by Treatment and Comparison Group eTable 5. Results from Sensitivity Analysis Using the RPO Data eFigure 1. Flow Chart of Patient-Year Observations Included in Analyses eFigure 2. Plots of Test of Differential Pre-Trends in Study Outcomes (Event Study Regression Results) [file jamahealthforum-e232875-s001.pdf]

Supplementary Online Content

Sinaiko AD, Curto VE, Ianni K, Soto M, Rosenthal MB. Utilization, steering, and spending in vertical relationships between physicians and health systems. *JAMA Health Forum.* 2023;4(9):e232875. doi:10.1001/jamahealthforum.2023.2875

eAppendix. Data Information

eMethods

eReferences

eTable 1. Classification of Health Systems

eTable 2. Hospitalization Characteristics

eTable 3. Patient-Year Characteristics

eTable 4. Physician Characteristics, Stratified by Treatment and Comparison Group

eTable 5. Results from Sensitivity Analysis Using the RPO Data

eFigure 1. Flow Chart of Patient-Year Observations Included in Analyses

eFigure 2. Plots of Test of Differential Pre-Trends in Study Outcomes (Event Study Regression Results)

This supplementary material has been provided by the authors to give readers additional information about their work.

© 2023 Sinaiko AD et al. *JAMA Health Forum.*

eAppendix. Data Information

The Massachusetts Provider Database

The Massachusetts Provider Database (MPD) is compiled by Massachusetts Health Quality Partners (MHQP). The MPD collects provider network annually directly from health plans and provider organizations (rather than ascertained from payer records), and reports each physician's practice site(s), medical group(s), and health system (if any). Approximately 95% of provider organizations validate the MPD annually using an online validation tool. There is high compliance because the provider hierarchy is used for the MHQP's annual statewide adult and child patient experience survey, and survey data are publicly released on a consumer-facing website. The survey is also partially funded by health plans, which use the survey results in their risk contracts with provider networks.[1]

The Center for Health Information and Analysis Data

The Center for Health Information and Analysis (CHIA) is an independent state agency in Massachusetts that collects financial and patient-level data from payers and providers in the state which are used by state agencies, researchers, providers, and payers. These datasets include the Massachusetts All-Payer Claims Database (MA APCD), hospital cost reports, health system financial performance, acute hospital case mix databases, and other cost reports for non-hospital providers.[2] In this study, we use the hospital cost reports, relative price data, and the MA APCD from CHIA.

Hospital Cost Reports and Relative Price

The hospital cost reports are collected annually and contain fields from the Medicare hospital cost reports (CMS 2552-10 filing) as well as state-specific information. The cost report includes information on payer mix, utilization, cost, and revenue.[3] We used years 2013, 2015, and 2017 to link hospitals in our sample to their respective health systems. The hospital cost report data are publicly available.[4] The CHIA relative price data are collected annually and are used to compare average provider prices across the state controlling for patient acuity, type of service, and insurance product. At the time of analysis, 2018 was the most recent year of relative price data available for use. The dataset includes both payer-specific and statewide relative prices to facilitate comparisons within and across payers. The relative price data are publicly available.[5]

Massachusetts All-Payer Claims Database

MA APCD data are reported directly by insurers within 30 days after the end of each month as part of a state mandate.[6] However, per the Supreme Court decision in *Gobeille v. Liberty Mutual* in 2016, employers who offer self-funded plans could decline to submit data to the APCD.[7] This had an impact on the MA APCD; at the end of 2017, approximately 75% of self-insured individuals in Massachusetts were no longer in the MA APCD.[8] In our study sample for years 2013-2017, self-insured plans respectively account for 48.2%, 49.4%, 48.9%, 37.3%, and 35.9% of beneficiaries in the enrollment files. Because not all enrolled have medical claims, we also assessed the share of self-insured plans with any medical expenditures in our study sample for years 2013-2017 and found the shares to be 41.8%, 44.0%, 42.3%, 20.1%, and 18.5%, respectively.

© 2023 Sinaiko AD et al. *JAMA Health Forum.*

We obtained the 2013-2017 MA APCD, which was the most recent five-year period available in 2020 when we began this project. The APCD includes seven file types: Medical Claims, Pharmacy Claims, Dental Claims, Member Eligibility, Provider, Product, and the Benefit Plan Control Total File as well as a separate enhanced eligibility file from MassHealth.[9] Each month, CHIA conducts data quality assurance and notifies payers if their files require adjustments. Each year prior to publishing the APCD, CHIA performs a quality assurance test to validate the completeness and quality of select fields. The claims files cover medical, pharmacy, dental, vision, behavioral health, and specialty services. The member eligibility file includes a unique individual identifier as well as member demographics, race, ethnicity, plan type, enrollment start and end dates, and other fields.

© 2023 Sinaiko AD et al. *JAMA Health Forum.*

eMethods

Health System Classification

We used the CHIA hospital reports to identify the hospitals in each health system. Using the hospital reports that contained the name of the health system that the hospital was part of, we constructed a comprehensive list of hospitals associated with each health system. We focused on the 10 health systems in both the MHQP and CHIA data sources, which were each affiliated with at least 2 and up to 8 hospitals. The health systems varied in the share of hospital discharges within the health system's geographic market based on hospital referral region (HRR) in 2017 (eTable 1).

A system was classified as large if the share of total patient discharges from all hospitals within the system was at least 20% of the health system's geographic market, defined using the HRR in 2017 (eTable 1).

Vertical Relationship Classification

We developed a novel measure of a "vertical relationship" between a PCP and hospitals that captures full integration, or outright ownership, as well as joint contracting, or affiliation. We defined a physician as "vertically related" with one of the large health systems described in eTable 1, if the physician was classified as being in that health system according to the MHQP master provider database. In addition, for approximately 5% of our sample of physician-year observations we manually reviewed medical groups that were not part of any system according to the MHQP data and determined whether they were affiliated with any hospital through a combination of reviewing whether the medical group's website mentioned a hospital affiliation and whether the medical group's location was shown to be at a hospital according to Google Maps. For these medical groups, we defined their physicians as having a "vertical relationship" with a health system if the medical group was affiliated with a hospital that was part of a health system according to the CHIA data. We classified physicians who were part of the Atrius network, according to the MHQP data, as independent.

Wellforce is a health system that was established in 2014 and included a network of physicians called the New England Quality Care Alliance (NEQCA). We defined no physicians as vertically integrated or joint contracting with Wellforce in 2013, since it was not yet established; and we defined physicians who were part of the NEQCA network, according to the MHQP data, as vertically related with Wellforce in 2015 and 2017.

We note that our study diverges from other studies in that we measure vertical relationships as opposed to vertical integration/outright ownership, since we rely on physician affiliations collected by MHQP. For instance, a number of other studies including Capps, Dranove, and Ody (2018)construct a measure of vertical integration in part by using tax identification numbers (TINs).[10] Although such a measure likely captures many ownership relationships, it is complicated by the fact that physicians may bill under multiple TINs. When a system or hospital acquires a practice, it may consolidate TINs, but there is no requirement to do so.[11] Notably, Capps, Dranove, and Ody (2018) supplement their use of TINs by incorporating information from SK&A data, which contain information about health care organizational structure similar to that in the MHQP data. While the MHQP data does not differentiate between ownership and affiliation, it is possible to identify affiliation and ownership using physician

roster files from the Massachusetts Health Policy Commission's Registered Provider Organization database, as done in a previous study.[12] Curto, Sinaiko, and Rosenthal (2022) found that Baycare, Berkshire, Heywood, Lahey, Partners, UMass and Wellforce have a higher share of corporate affiliations than contracting affiliations while Beth Israel, Cape Cod, and Steward have a higher share of contracting affiliations than corporate affiliations.[12]

Attribution of Primary Care Physicians

First, physicians were identified as in one of three categories: Primary Care Physician (PCP), Specialist, or Other, in the MHQP data. We linked physicians to the MA APCD data using National Provider Identifier (NPI) number from MHQP data. We identified patients in the MA APCD with commercial insurance from an included carrier, at least one E&M claim in a given year, and who were enrolled for at least seven months.

We attributed this sample of patients to PCPs based on the plurality of E&M claims in the patient-year in which patient outcomes are measured using previously defined patient attribution methods.[13] First, the number of E&M claims associated with each NPI was totaled at the patient-year level. The PCP with the most E&M claims was attributed to that patient-year. If a patient had equal numbers of E&M claims with more than one PCP (12.3% of attributed patient-years), we randomly assigned the patient to one of the PCPs (the one with the lower NPI). Patients with no E&M claims with a PCP (12.9% of attributed patient-years) were attributed to the physician associated with the plurality of their E&M claims (e.g., from another specialty, hospitalist). We conducted sensitivity analyses where we excluded these patient-years and results were similar. We also gave preference to NPIs that were present in the MHQP; when the plurality of claims was with a non-MHQP NPI, we assigned that patient-year to the NPI in MHQP with the most claims (11.0% attributed patient-years). We conducted sensitivity analyses where we excluded these patient-years and results were similar.

Study Variables

Hospitalizations. Our inpatient hospitalization rates generally align with reports of annual discharges for commercially insured patients in Massachusetts from other sources. For 2016, we reported 28,921 discharges for 687,530 commercially insured patients in our sample (42.1 hospitalizations per 1,000 enrollees). CHIA reported 250,695 inpatient discharges in 2016 for 4,117,315 (expected) commercially insured patients (60.9 per 1,000 enrollees), representing about 31% of discharges in the state.[14] The Massachusetts Health Policy Commission reported 55.6 commercially insured discharges per 1,000 residents in 2016.[15] Our discharge rate may be slightly lower than reported rates because as of 2016, the APCD does not contain all claims from self-insured employers.

Hospital Price Terciles. We used CHIA's relative price information to construct hospital price terciles.[16] CHIA calculated relative prices for a payer's network, defined as a unique provider-insurance type combination (e.g., Acute Inpatient Hospital-Commercial). For hospital relative prices, CHIA computed the hospital-specific aggregate price levels, taking an unweighted average of hospital-specific price levels to calculate the network average price level, and dividing hospital-specific price level by network average price level to obtain each hospital's relative price. By construction, the network average relative price equals 1.0 for each network

therefore hospitals with a relative price above 1.0 received higher-than-average payments.[17] We measured relative costliness of hospital care by grouping all hospitals in Massachusetts into terciles using the CHIA hospital-commercial payer relative price index (eTable 2).

Patient Clinical Risk. We measured patient clinical risk using Hierarchical Condition Category (HCC) codes per patient-year. HCC codes were developed for the CMS Medicare Advantage program[18] and are used in the individual and small group marketplace to predict medical expenditure risk based on patient diagnoses and demographics.[19] First, we identified HCCs using diagnosis codes reported on the claims. Then, we constructed binary variables that indicate if a patient had 0, 1, 2, 3, 4, or 5 or more HCC codes in a given year, where more is indicative of higher clinical risk (eTable 3).

Market Concentration. Because vertical integration may also have a horizontal component (e.g., if a system that already owns physician groups acquires additional physicians, both vertical market concentration and horizontal market concentration increase) and we wanted to isolate the vertical effects of vertical integration, we controlled for market concentration among physicians by specialty using the Herfindahl-Hirschman Index (HHI) at the 3-digit ZIP code level. We calculated HHIs following a method used in Capps, Dranove, and Ody (2018)[10] and Kessler and McClellan (2000).[20] We started by calculating HHIs for each patient 3-digit ZIP code (the most granular geographic unit observed in our version of the MA APCD), specialty, and year combination. We then assigned an HHI to a physician by calculating the weighted average of the HHIs for the 3-digit ZIP codes that she serves. The weights were calculated using the physician's firm's share of spending across the 3-digit ZIP codes it serves.

Comparison Group

In a stacked event study, comparing the pre-, untreated-period of a later-treated group to the post-period of an early-treated group is problematic.[21] Our paper does not include that comparison in any of our analyses. There are tradeoffs associated with the choice of comparison group units, and the key methodological piece is that the comparison group is justified with the appropriate conditional parallel trends assumptions.[21,22]

Our comparison groups included only patients whose PCP was either never in a vertical relationship ("never-treated") or always in a vertical relationship ("always-treated") with a large system from 2013-17 (eTable 4). Although there are concerns with using "always-treated" units in the comparison group,[49] we included these observations because the always-treated physicians are plausibly more similar to the treatment group in that they also selected to join vertical relationships with health systems. In addition, this paper examines both total utilization and within-system utilization (steering) and the "never-treated" physicians do not have a measure of within-system specialist visits (because they are not vertically related with any system, we cannot measure within-system visits). Therefore, the "never-treated" units cannot be used as a comparison for the steering outcomes. In contrast, the "always-treated" units allow us to compare the within-system utilization of VI physician-patient pairs before versus after they enter vertical relationships to the within-system utilization patterns of physician-patient pairs that are always part of large health systems. We view this as somewhat of a different association parameter (or as Callaway and Sant'Anna put it, "policy-relevant disaggregated causal

© 2023 Sinaiko AD et al. *JAMA Health Forum.*

parameters") [21]rather than a biased estimate of the association between vertical relationships and outcomes. In a summary of the literature by Chaisemartin and D'Haultfoeuille (2022)[23], the authors note that including the "always-treated" group in a comparison is necessary but not sufficient to have negative weights. In other words, using "always-treated" units could result in negative weights but do not automatically imply negative weights. An approach to investigating the weighting is through comparison of estimates with and without "always-treated" units. In sensitivity analyses, we tested whether the results were robust to inclusion of the "always-treated" units in the comparison group by estimating our empirical models using only the "never-treated" physicians as the comparison group, for all outcomes except the within-system utilization measures. The results from this sensitivity analysis are similar to the main study results.

We tested for and found support for parallel trends in outcomes between the treatment and the pooled comparison groups in the pre-period (eFigure2). In addition, to assess the appropriateness of including the "always-treated" units we looked at trends in the evolution of the outcome between treated and the never-treated group, and between treated and the always-treated group, for all outcomes except the within-system utilization measures. (For the within-system utilization measures the comparison group only includes the always-treated physicians and the pre-trends were parallel.) For all outcomes except one the always-treated and never-treated groups have the same trends. For the total specialists visits outcome the pre-period trend among the always treated group is slightly different than that for the never-treated group. Use of never-treated alone versus never-treated and always-treated as a pooled comparison group for this outcome (total specialist visits) did not significantly affect our estimates.

Analysis

We used the following specification for our main results:

$$Y_{ipts} = \lambda(treat_{ps} * post_{st}) + \gamma_s + \delta_t + \alpha_p + \mathrm{B}'\boldsymbol{X}_{ipt} + \varepsilon_{ipts} \qquad (1)$$

Y_{ipts} is the outcome for patient i attributed to physician p in year t in experiment s, $treat_{ps}$ indicates if physician p in experiment s is in a vertical relationship with a large system and $post_{st}$ indicates that the physician p in experiment s is in the post-period in calendar year t. Experiment s includes 2015 and 2017. The coefficient of interest, λ, can be interpreted as the average change in the outcome associated with a patient's attributed physician becoming vertically integrated with a large health system.

We included fixed effects for experiment (γ_s), year (δ_t), and physician (α_p). The term $\boldsymbol{X}_{ipt}$ represents a vector of covariates including market concentration at the 3-digit ZIP code level using the HHI, patient clinical risk using HCC bins, the number of months an individual is continuously enrolled in health insurance, age bins, patient sex, the interaction of age and sex, and patients with no sex reported in the APCD. Because physicians are organized into medical groups and entire medical groups may become vertically integrated at the same time, standard errors were clustered at the medical group level. Medical groups are parent provider organizations that are comprised of multiple physicians and may include multiple practices and practice sites. Medical groups are obtained from the MHQP data, which contain physician lists

© 2023 Sinaiko AD et al. *JAMA Health Forum.*

that are organized into a hierarchy from physician to practice site to medical group to health system.

We estimated a version of Equation 1 with the addition of an interaction between experiment fixed effects and physician fixed effects. The results were nearly identical to those from Equation 1, so we do not include them here.

Sensitivity Analysis Using the RPO Data

We obtained the 2015 and 2017 physician roster files from the Massachusetts Health Policy Commission's Registered Provider Organization (RPO) database to better understand the relationships between provider organizations and physician practices. The physician roster file does not exist for the other years of our sample period. These files are not publicly available but were provided to us by the Massachusetts Health Policy Commission.

We combined the physician roster file with a publicly available RPO file listing all registered provider organizations and their corporate and contracting affiliates. As the RPO documentation clarifies, "affiliates tend to be direct providers of patient care… these include, but are not limited to, solo practitioners, physician groups, hospitals, and community health centers." We used an "organization type" data field to limit the list of affiliates to physician practices. The RPO documentation also clarifies that "corporate affiliates are owned or controlled by the identified provider organization… contracting affiliates are entities that the provider organization negotiates contracts on behalf of but does not own or control." We can therefore consider "corporate affiliation" to indicate ownership.

For physicians in the physician roster file, the medical group usually coincided with the affiliate name in the RPO file. We also matched medical groups to affiliate names that were nearly identical except for small discrepancies such as obvious abbreviations used in one file but not the other, or an "Inc." or "LLC" ending used in one file but not the other. Note that a medical group could appear in the physician roster file but not in the RPO file if the medical group was not a corporate affiliate or contracting affiliate of any registered provider organization. It would also not appear if there was a discrepancy in the medical group name such that it did not match with the affiliate name in the RPO file, though we manually reviewed all affiliate names in the RPO file to match as many as possible.

For the sample of physicians in our MHQP data set from 2015 and 2017, we were able to compare their system affiliations according to the MHQP data with the corporate and contracting affiliations according to the RPO files from corresponding years. Among physicians in the MHQP sample, 94.1% were matched to a physician in the RPO physician roster file using the physician NPI. Although this comparison is imperfect because of the difficulties in matching physicians and medical groups across the two data sets, it does strongly suggest that the affiliations in the MHQP data indicate a combination of ownership and joint contracting.

We implemented a sensitivity analysis focusing on a stricter definition of vertical integration that only corresponds to ownership, or the corporate affiliations according to the RPO data. We excluded physicians who entered vertical relationships in 2015 or 2017 that were not corporate affiliations according to the RPO data. This was to exclude physicians who were joint

© 2023 Sinaiko AD et al. *JAMA Health Forum.*

contracting from our treatment group. We then estimated our main specifications using this strict ownership definition of vertical integration. Results are shown in eTable 5. These results are consistent with our findings for the broader sample but estimated associations are slightly larger in magnitude.

© 2023 Sinaiko AD et al. *JAMA Health Forum.*

eReferences

1. Massachusetts Health Quality Partners (MHQP). MA Provider Directory (MPD). Accessed April 2, 2022. https://www.mhqp.org/resources/massachusetts-provider-directory-mpd/

2. Center for Health Information Analysis (CHIA). Hospital and Other Provider Data. Hospital and Other Provider Data. Accessed April 2, 2022. https://www.chiamass.gov/data-index/

3. Center for Health Information Analysis (CHIA). Information for Data Submitters: Hospital Cost Reports. Accessed April 2, 2022. https://www.chiamass.gov/hospital-cost-reports-2/

4. Hospital Cost Report External Use Registration. Accessed April 21, 2022. https://www.chiamass.gov/hospital-cost-report-external-use/

5. Relative Price and Provider Price Variation. Accessed April 21, 2022. https://www.chiamass.gov/relative-price-and-provider-price-variation/

6. Center for Health Information Analysis (CHIA). Massachusetts All-Payer Claims Database. Accessed March 27, 2022. https://www.chiamass.gov/ma-apcd/

7. Center for Health Information Analysis (CHIA). Key Regulatory Issues Facing APCD States post Gobeille v. Liberty Mutual. https://www.chiamass.gov/assets/docs/p/apcd/regulatory-questions-for-apcds-related-to-scotus.pdf

8. Hobbs SD, Medinus A. DEMOGRAPHIC DIFFERENCES IN MASSACHUSETTS ALL PAYER CLAIMS DATA (MA APCD) BEFORE AND AFTER GOBEILLE. Presented at: https://www.nahdo.org/sites/default/files/2020-08/Day%20Four%20Slides/402-70%20Sylvia%20Hobbs%20NAHDO_August2020_HOBBS_MEDINUS.pdf

9. Center for Health Information Analysis (CHIA). *OVERVIEW OF THE MASSACHUSETTS ALL-PAYER CLAIMS DATABASE.*; 2016. https://www.chiamass.gov/assets/docs/p/apcd/APCD-White-Paper-2016.pdf

10. Capps C, Dranove D, Ody C. The Effect of Hospital Acquisitions of Physician Practices on Prices and Spending. *Journal of Health Economics*. 2018;59:139-152.

11. Welch WP, Cuellar AE, Stearns SC, Bindman AB. Proportion of Physicians in Large Group Practices Continued to Grow in 2009-11. *Health Affairs*. 2013;32(9):1659-1666. doi:10.1377/hlthaff.2012.1256

12. Curto V, Sinaiko AD, Rosenthal MB. Price Effects Of Vertical Integration And Joint Contracting Between Physicians And Hospitals In Massachusetts. *Health Affairs*. 2022;41(5):741-750.

13. Lewis VA, McClurg AB, Smith J, Fisher ES, Bynum JPW. Attributing Patients To Accountable Care Organizations: Performance Year Approach Aligns Stakeholders' Interests. *Health Affairs*. 2013;32(3):587-595. doi:10.1377/hlthaff.2012.0489

© 2023 Sinaiko AD et al. *JAMA Health Forum.*

14. CHIA. *Massachusetts Discharge Data: Hospital Inpatient Acute Care.*; 2020.

15. Massachusetts Health Policy Commission. 2018 Annual Health Care Cost Trend Report. Published online 2019.

16. Center for Health Information Analysis (CHIA). Relative Price and Provider Price Variation. Accessed March 27, 2022. https://www.chiamass.gov/relative-price-and-provider-price-variation/

17. Center for Health Information Analysis (CHIA). *Relative Price and Provider Price Variation in the Massachusetts Commercial Market: Technical Appendix.*; 2021. https://www.chiamass.gov/assets/docs/r/pubs/2021/Relative-Price-Technical-Appendix-2019.pdf

18. Pope GC, Kautter J, Ellis RP, et al. Risk Adjustment of Medicare Capitation Payments Using the CMS-HCC Model. *Health Care Financ Rev*. 2004;25(4):119-141.

19. Kautter J, Pope GC, Ingber M, et al. The HHS-HCC Risk Adjustment Model for Individual and Small Group Markets under the Affordable Care Act. *Medicare Medicaid Res Rev*. 2014;4(3):mmrr2014-004-03-a03. doi:10.5600/mmrr.004.03.a03

20. Kessler DP, McClellan MB. Is Hospital Competition Socially Wasteful? *The Quarterly Journal of Economics*. 2000;115(2):577-615.

21. Callaway B, Sant'Anna PHC. Difference-in-Differences with multiple time periods. *Journal of Econometrics*. 2021;225(2):200-230. doi:10.1016/j.jeconom.2020.12.001

22. Sun L, Abraham S. Estimating dynamic treatment effects in event studies with heterogeneous treatment effects. *Journal of Econometrics*. 2021;225(2):175-199. doi:10.1016/j.jeconom.2020.09.006

23. de Chaisemartin C, D'Haultfœuille X. Two-Way Fixed Effects Estimators with Heterogeneous Treatment Effects. *American Economic Review*. 2020;110(9):2964-2996. doi:10.1257/aer.20181169

© 2023 Sinaiko AD et al. *JAMA Health Forum.*

eTable 1. Classification of Health Systems

System	Classification (1)	Share of Patient Hospital Discharges (%) Boston HRR (2)	Springfield HRR (3)	Worcester HRR (4)	State of MA (5)	# of Hospitals State of MA (6)
Baycare	Large	0.0	53.5	0.0	6.5	4
Berkshire	Medium	0.0	16.1	0.0	2.0	2
Cape Cod	Small	3.6	0.0	0.0	2.8	2
Beth Israel	Medium	13.1	0.0	0.0	10.1	6
Heywood	Small	0.0	0.0	0.0	0.0	2
Lahey	Small	9.1	0.0	0.0	7.1	3
Partners	Large	23.8	7.1	0.0	19.3	8
Steward	Medium	12.2	0.0	0.0	9.5	8
UMass	Large	0.0	0.0	64.9	6.7	4
Wellforce	Small	8.1	0.0	0.0	6.3	3
None	Independent	30.0	23.3	35.1	29.7	--
Observations		631,677	99,385	83,737	814,799	--

Authors' analysis of Massachusetts Health Quality Partners (MHQP) provider data 2013, 2015, and 2017 and Center for Health Information and Analysis (CHIA) Massachusetts hospital cost reports 2013, 2015, and 2017
NOTES: HRR = Hospital Referral Region
The table shows the share of patient hospital discharges as well as the number of hospitals in each health system in Massachusetts in 2017. The share of patient hospital discharges is shown separately for each of the HRRs in Massachusetts. Cells highlighted in red indicate that a system's share in an HRR is greater than or equal to 20%. Column 1 shows the classification of systems.

© 2023 Sinaiko AD et al. *JAMA Health Forum.*

eTable 2. Hospitalization Characteristics

		Mean	SD	10th Percentile	90th Percentile
Hospital Prices	Proportion of patient-years with hospitalizations at facilities with non-missing relative price data	0.04	0.18	0	0
	Admission to Lowest Prices Tercile 1 (0.66-0.86, n=21)	0.17	0.38	0	1
	Admission to Middle Prices Tercile 2 (0.87-1.01, n=20)	0.30	0.46	0	1
	Admission to Highest Prices Tercile 3 (1.01-1.92, n=20)	0.54	0.50	0	1
Readmission Rates	Proportion of patient-years with hospitalizations at facilities with non-missing all-cause 30-day readmissions data	0.03	0.17	0	0
	Mean hospital-wide readmission rate	16.43	1.07	16	18
	Admission to Lowest Readmissions Tercile 1 (11.8%-15.8%, n=22)	0.23	0.42	0	1
	Admission to Middle Readmissions Tercile 2 (15.9%-16.4%, n=15)	0.24	0.42	0	1
	Admission to Highest Readmissions Tercile 3 (16.5%-17.7%, n=16)	0.55	0.50	0	1
Observations		4,030,224			

Authors' analysis of Massachusetts Health Quality Partners (MHQP) provider data 2013, 2015, and 2017, All Payer Claims Data (APCD), 2013-17, Center for Health Information and Analysis (CHIA) Provider Relative Price data 2018, and CMS Hospital Care Compare hospital-wide readmissions rates 2018.

© 2023 Sinaiko AD et al. *JAMA Health Forum.*

eTable 3. Patient-Year Characteristics

	Mean	SD	10th Percentile	90th Percentile
Months enrolled	10.86	1.76	8	12
Patient age	35.07	19.95	6	60
Female	53.3%	49.9%	0.0%	100.0%
Age 0 - 17, male	13.4%	34.1%	0.0%	100.0%
Age 18 - 30, male	6.2%	24.1%	0.0%	100.0%
Age 31 - 40, male	5.6%	23.0%	0.0%	100.0%
Age 41 - 50, male	7.8%	26.9%	0.0%	0.0%
Age 51 - 64, male	12.1%	32.6%	0.0%	0.0%
Age 65+, male	1.6%	12.5%	0.0%	0.0%
Age 0 - 17, female	12.9%	33.5%	0.0%	100.0%
Age 18 - 30, female	8.5%	27.8%	0.0%	0.0%
Age 31 - 40, female	7.3%	26.0%	0.0%	100.0%
Age 41 - 50, female	9.5%	29.3%	0.0%	0.0%
Age 51 - 64, female	13.8%	34.5%	0.0%	0.0%
Age 65+, female	1.4%	11.7%	0.0%	0.0%
Missing Gender	0.0%	0.2%	0.0%	100.0%
0 HCCs	68.7%	46.4%	0.0%	100.0%
Any HCC	31.3%	46.4%	0.0%	100.0%
1 HCC	19.6%	39.7%	0.0%	100.0%
2 HCCs	7.1%	25.7%	0.0%	0.0%
3 HCCs	2.5%	15.8%	0.0%	0.0%
4 HCCs	1.0%	10.1%	0.0%	0.0%
5+ HCCs	1.1%	10.5%	0.0%	0.0%
Observations	4,030,224			

Authors' analysis of Massachusetts Health Quality Partners (MHQP) provider data 2013, 2015, and 2017 and All Payer Claims Data (APCD), 2013-17.

NOTES: HCC = Hierarchical Condition Category; SD = Standard Deviation. Values represent the mean share across patient-years. For example, in our multi-year analytic sample, 53.3% of patients are female.

© 2023 Sinaiko AD et al. *JAMA Health Forum.*

eTable 4. Physician Characteristics, Stratified by Treatment and Comparison Groups

	All		Treatment Group		Always-Treated Physicians		Never-Treated Physicians	
	**Mean**	**SD**	**Mean**	**SD**	**Mean**	**SD**	**Mean**	**SD**
**Physician Characteristics**								
Physician Market HHI[a]	0.30	0.12	0.29	0.12	0.34	0.14	0.27	0.09
# Patients Assigned to Medical Group	100,495.88	72,448.70	79,225.14	54,990.97	125,865.63	67,856.22	81,391.32	70,584.40
**Characteristics of Physician's Patients**								
Months enrolled	10.73	0.78	10.67	0.65	10.74	0.87	10.72	0.70
Age	39.18	13.67	39.72	12.16	39.80	13.72	38.65	13.70
Female	0.53	0.26	0.52	0.24	0.54	0.28	0.53	0.26
Age 0 - 17, male	0.07	0.16	0.06	0.14	0.06	0.16	0.07	0.16
Age 18 - 30, male	0.08	0.13	0.09	0.12	0.08	0.14	0.09	0.12
Age 31 - 40, male	0.07	0.11	0.07	0.08	0.07	0.12	0.07	0.11
Age 41 - 50, male	0.09	0.12	0.10	0.12	0.08	0.13	0.09	0.12
Age 51 - 64, male	0.13	0.17	0.14	0.16	0.13	0.18	0.13	0.16
Age 65+, male	0.02	0.07	0.02	0.05	0.03	0.08	0.02	0.07
Age 0 - 17, female	0.07	0.16	0.06	0.14	0.06	0.15	0.07	0.16
Age 18 - 30, female	0.11	0.15	0.10	0.11	0.11	0.15	0.11	0.15
Age 31 - 40, female	0.09	0.14	0.09	0.11	0.10	0.15	0.09	0.12
Age 41 - 50, female	0.10	0.13	0.11	0.12	0.10	0.14	0.10	0.12
Age 51 - 64, female	0.15	0.17	0.14	0.12	0.15	0.18	0.14	0.15
Age 65+, female	0.02	0.07	0.02	0.03	0.02	0.07	0.02	0.07
Missing Gender	0.00	0.00	0.00	0.00	0.00	0.00	0.00	0.00
1+ HCCs[b]	0.44	0.29	0.41	0.26	0.49	0.31	0.40	0.26
0 HCCs	0.56	0.29	0.59	0.26	0.51	0.31	0.60	0.26
1 HCC	0.22	0.18	0.22	0.16	0.23	0.20	0.22	0.17
2 HCCs	0.10	0.14	0.09	0.11	0.11	0.16	0.10	0.13
3 HCCs	0.05	0.11	0.04	0.11	0.06	0.12	0.04	0.09
4 HCCs	0.02	0.08	0.02	0.03	0.03	0.10	0.02	0.07
5+ HCCs	0.04	0.14	0.04	0.15	0.06	0.17	0.03	0.11

© 2023 Sinaiko AD et al. *JAMA Health Forum.*

16,406	484	7,071	8,851

Authors' analysis of Massachusetts Health Quality Partners (MHQP) provider data 2013, 2015, and 2017 and All Payer Claims Data (APCD), 2013-17.

NOTES PCP = primary care physician; ED = Emergency Department.

[a] Defined as the physician market Herfindahl-Hirschman Index (HHI) of total patient spending at the ZIP3 level.

[b] Chronic conditions are measured using Hierarchical Condition Category (HCC) codes, developed for the CMS Medicare Advantage program and used in the individual and small group marketplace to predict medical expenditure risk based on patient diagnoses and demographics.

eTable 5. Results from Sensitivity Analysis Using the RPO Data

	Sample Size	Comparison group mean (2013-17)	Estimated coefficient	95% CI	p-value	Percent change over comparison group mean
**Spending outcomes**						
Total Medical Expenditure	7,593,967	5,700.07	611.63	122.14 to 1,101.11	0.01	10.73%
Probability of Admission to a High-Price Hospital	266,072	0.55	0.07	0.02 to 0.11	0.006	11.98%
**Readmissions outcomes**						
Readmission Rate	281,465	0.06	0.00	-0.01 to 0.02	0.57	6.48%
Probability of Admission to a Low Readmission Rate Hospital	237,926	0.23	0.03	-0.02 to 0.07	0.24	11.26%
**Utilization Outcomes**						
Specialist Visits	7,593,967	3.06	0.68	0.02 to 1.35	0.04	22.28%
ED Visits	7,593,967	0.36	-0.02	-0.03 to 0.00	0.05	-4.82%
Hospitalizations	7,593,967	0.04	0.00	0.00 to 0.01	0.36	3.57%
**Within-System (Steering) Outcomes**						
Specialist Visits	2,900,574	2.73	1.12	0.73 to 1.51	<0.001	40.89%
ED Visits	2,900,574	0.15	0.03	0.02 to 0.05	<0.001	21.77%
Hospitalizations	2,900,574	0.03	0.01	0.00 to 0.02	<0.001	31.63%

Authors' analysis of Massachusetts Health Policy Commission's Registered Provider Organization (RPO) provider data 2015 and 2017, and All Payer Claims Data (APCD), 2013-17.
NOTES PCP = primary care physician.

Vertical relationships are defined as vertically integrated or joint contracting between a physician's medical group with a large health system. "Large" systems are those with the share of hospital discharges of at least 20% of the system's hospital referral region in 2017. Results based on stacked event study models including patient characteristics, experiment, year and physician fixed effects, and the physician market Herfindahl-Hirschman Index (HHI) of total patient spending at the ZIP3 level. Robust standard errors are clustered by medical group.

eFigure 1. Flow Chart of Patient-Year Observations Included in Analyses

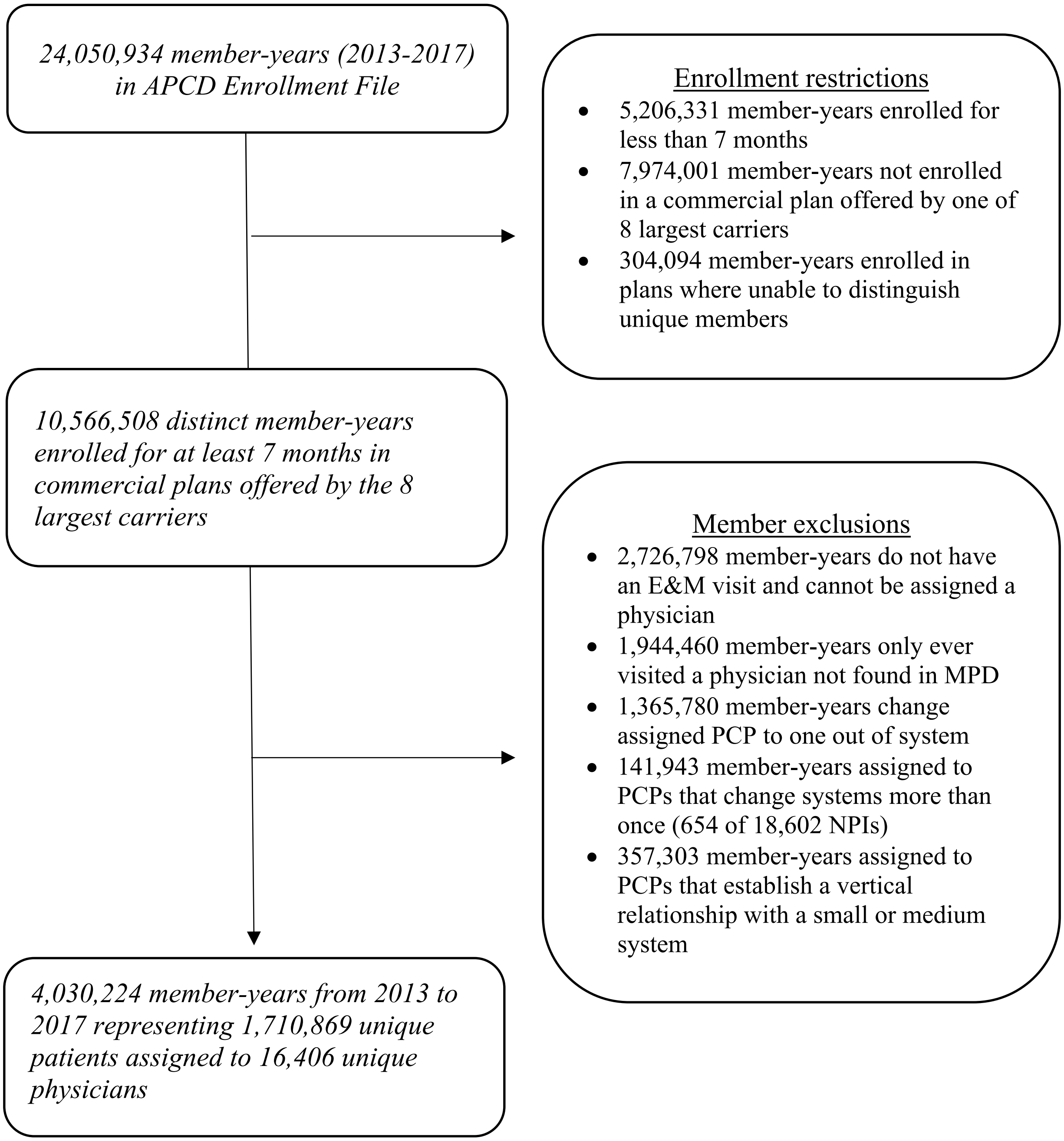

© 2023 Sinaiko AD et al. *JAMA Health Forum.*

eFigure 2. Plots of Test of Differential Pre-Trends in Study Outcomes (Event Study Regression Results)

Total Within-System Specialist Visits

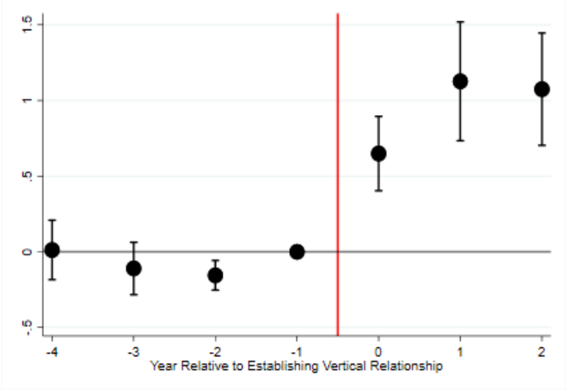

Total Specialist Visits

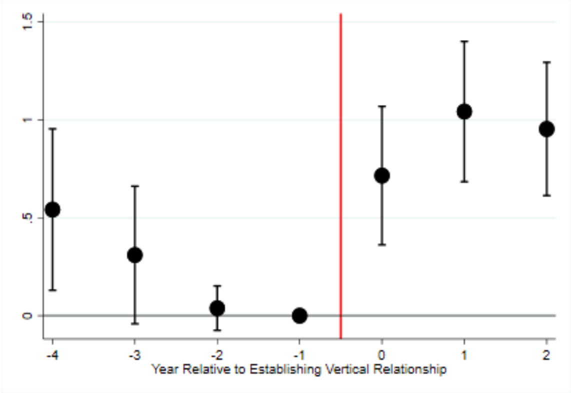

Total Within-System Hospitalizations

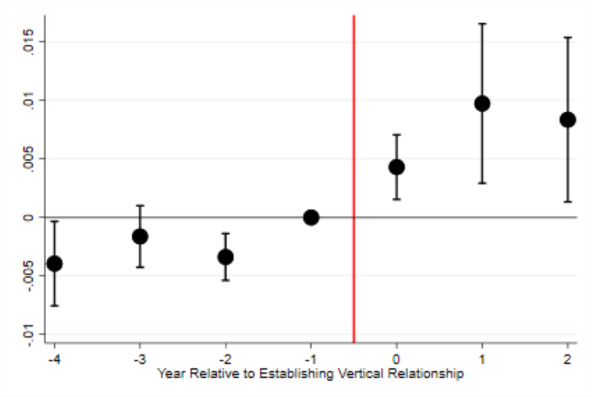

Total Hospitalizations

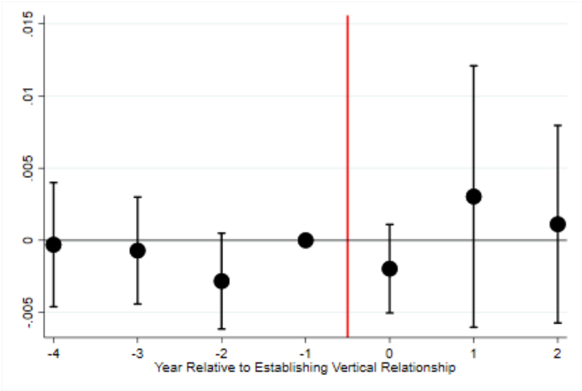

Total Within-System ED Visits

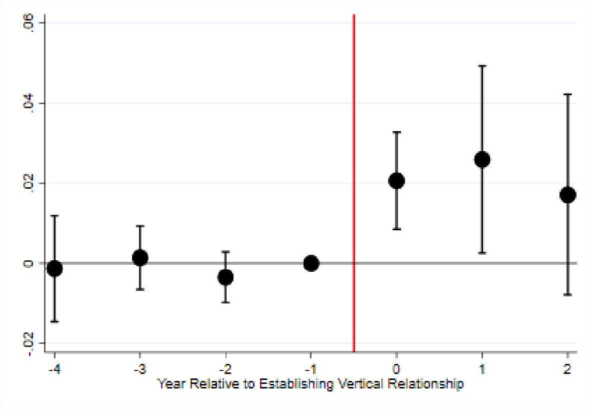

Total ED Visits

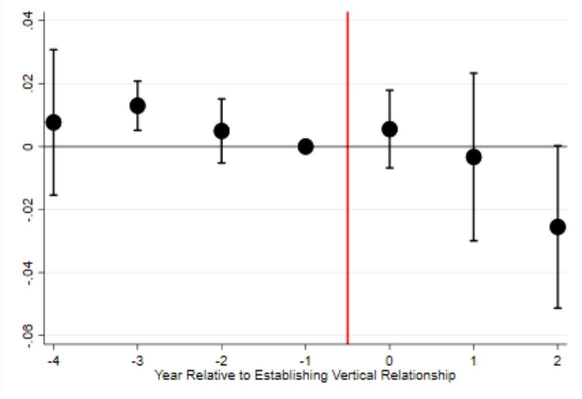

© 2023 Sinaiko AD et al. *JAMA Health Forum.*

Total Medical Expenditure

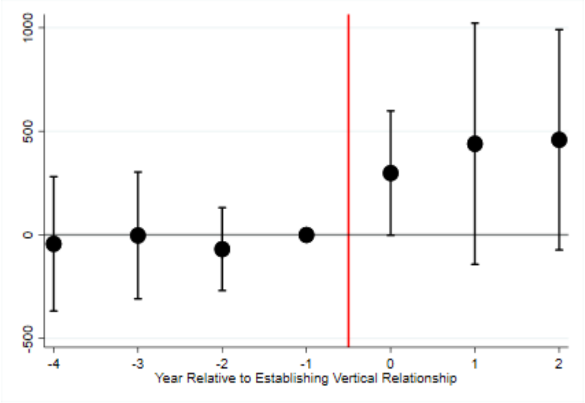

Probability of Admission to a High-Price Hospital

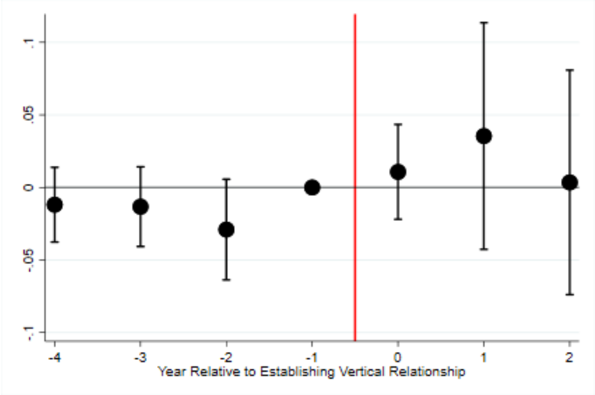

Probability of Admission to a Hospital with a Low All-Cause Readmission Rate

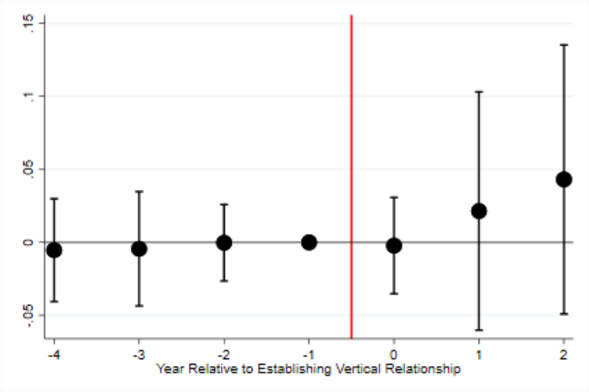

Probability of Readmission, Conditional on Inpatient Hospitalization

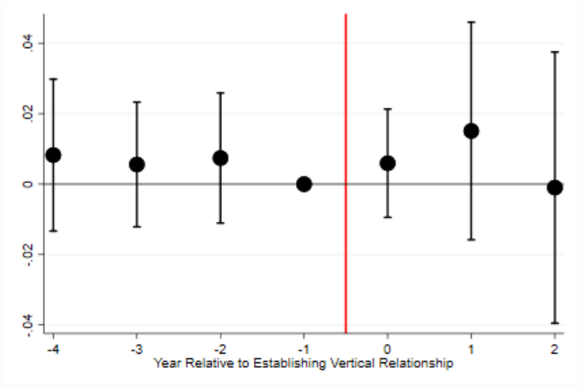

Authors' analysis of Massachusetts Health Quality Partners (MHQP) provider data 2013, 2015, and 2017 and All Payer Claims Data (APCD), 2013-17.

NOTES ED = Emergency Department. Results from event-study regression models where the last pre-treated year is the omitted category. Each sub-figure is from a separate regression. Bars represent 95 percent confidence intervals. Vertical line is at the year of establishing vertical relationship. Observations at the patient-year level. All models include the full study sample except for those testing the within-system steering measures which are limited to patients attributed to physicians in the treatment group or to physicians that had established vertical relationships prior to the study period (i.e. the "Always Treated" comparison group).

© 2023 Sinaiko AD et al. *JAMA Health Forum.*
