# Supplementary material for: Utilization, Steering, and Spending in Vertical Relationships Between Physicians and Health Systems
Source: JAMA Health Forum. 2023 Sep 1;4(9):e232875. doi: 10.1001/jamahealthforum.2023.2875 (PMC10474555; doi:10.1001/jamahealthforum.2023.2875)
Supplement: Supplement 2. — Data Sharing Statement [file jamahealthforum-e232875-s002.pdf]

Data Sharing Statement

Sinaiko. Utilization, Steering, and Spending in Vertical Relationships Between Physicians and Health Systems. *JAMA Health Forum*. Published September 01, 2023.
doi:10.1001/jamahealthforum.2023.2875

Data
Data available: No
